# Application of MnO_2_ Nanorod–Ionic Liquid Modified Carbon Paste Electrode for the Voltammetric Determination of Sulfanilamide

**DOI:** 10.3390/mi13040598

**Published:** 2022-04-10

**Authors:** Hadi Beitollahi, Somayeh Tajik, Antonio Di Bartolomeo

**Affiliations:** 1 Environment Department, Institute of Science and High Technology and Environmental Sciences, Graduate University of Advanced Technology, Kerman 7631885356, Iran; h.beitollahi@kgut.ac.ir; 2 Research Center of Tropical and Infectious Diseases, Kerman University of Medical Sciences, Kerman 7616913555, Iran; 3 Physics Department “E.R. Caianiello”, University of Salerno, Via Giovanni Paolo II 132, 84084 Fisciano, Italy

**Keywords:** MnO_2_ nanorod, ionic liquid, sulfanilamide, electrochemical sensor, voltammetry

## Abstract

The current work introduced a convenient single-phase hydrothermal protocol to fabricate MnO_2_ nanorods (MnO_2_ NRs). Fourier transform infrared spectroscopy (FT-IR), X-ray diffraction (XRD), Energy-dispersive X-ray spectroscopy (EDX) and field-emission scanning electron microscopy (FE-SEM) were used to determine the characteristics of MnO_2_ NR. Then, ionic liquid (IL) and MnO_2_ NRs were utilized to modify a carbon paste electrode (CPE) surface (MnO_2_NR-IL/CPE) to voltammetrically sense the sulfanilamide (SAA). An enhanced voltammetric sensitivity was found for the as-developed modified electrode toward SAA when compared with a bare electrode. The optimization experiments were designed to achieve the best analytical behavior of the SAA sensor. Differential pulse voltammetry (DPV) in the optimized circumstances portrayed a linear dependence on various SAA levels (between 0.07 and 100.0 μM), possessing a narrow detection limit (0.01 μM). The ability of the modified electrode to be used in sensor applications was verified in the determination of SAA present in the actual urine and water specimens, with impressive recovery outcomes.

## 1. Introduction

Sulfonamides (SAs) are agents that were able to show a selective effect on bacteria for the first time. They are systematically prescribed to control bacterial infections. The structure of sulfonamides contains an amino group bound with a phenyl ring having an alkyl sulfonamide in the para direction [1,2]. 4-aminobenzenesulfonamide, also called sulfanilamide or SAA, with the formula C_6_H_8_N_2_O_2_S is an antibiotic with antibacterial activity. The antibacterial response of SAA is related to the competitive inhibition of dihydropterase synthetase relative to paminobenzoate [3,4]. Despite the numerous benefits of antibiotics, they are potential contaminants. Hence, there are numerous reports of adverse effects on human health and the formation of resistant bacteria following long-term and over-administration of antibiotics. The biotic and abiotic degradability of SAA is weak in nature, so its residues can be found in various aquatic solutions like waste treatment plant effluents, surface water and groundwater, which is a public health problem worldwide [5,6]. This introduction highlights the need to take a robust approach to quantifying SAA at trace levels in different environments.

There are multiple accurate techniques for sensing the SAA, some of which are liquid chromatography [7], high performance liquid chromatography [8,9], spectrophotometry [10], chemiluminescence [11], and capillary electrophoresis-mass spectrometry [12]. However, some of these methods have disadvantages such as a high cost, a time-consuming operation, unsuitability for on-site analysis, and the need for qualified operators and complicated instrumentations.

Among all sensing systems, great attention has been focused on the electrochemical determinations owing to their impressive merits like simple use, affordability, portability, exceptional selectivity, and sensitivity and rapidity [13,14,15,16,17,18,19]. Voltammetry has had a great role in the detection of various analytes in environmental, biomedical and food matrices. Numerous studies reported the direct detection of analytes by voltammetry owing to accuracy, convenience, simplicity, sensitivity, and rapidity [20,21,22,23]. Hence, the SAA concentrations can also be detected by voltammetric methods. A major problem in this field is the need for a high overpotential due to the direct redox reaction of analytes on the bare electrode, as a result of which the appeared fouling impact leads to a weakness in reproducibility and selectivity [24,25].

Electrode surface modification can increase the sensitivity of the produced sensors due to the establishment of appropriate and adjustable features. Diverse modifiers in this field have offered a lower limit of detection (LOD), excellent sensitivity, overpotential reduction and surface fouling resistance [26,27,28,29,30,31,32].

The use of appropriate electrochemical methods in conjunction with carbon paste electrodes (CPEs) has yielded acceptable results. A variety of analytes have been detected by CPEs because of their unique properties, some of which include residual currents 10 times lower than glassy carbon electrodes (GCEs), a wider potential window, and facile preparation steps [33,34,35,36,37,38]. The selectivity and sensitivity of the target analyte determination can be significantly enhanced by chemically modified CPEs using modifiers [39,40,41,42].

One of the most successful modifiers used in electrochemical sensors is nanoparticles (NPs), and many special features of the working electrode, like the huge surface area and small size, reproducibility, peak current and sensitivity, can be enhanced in the presence of NPs [43,44].

Another modifiers are metal oxides, owing to their admirable traits, some of which include an adjustable production, huge specific surface area, direct paths for electron transfer, empty space to reduce volume expansion, proper adhesion of active substance to current collector and low cost [45,46]. A popular oxide material is manganese dioxide (MnO_2_), whose behavior can be enhanced by changing its morphology and surface area. MnO_2_ is a polymorph (1D α-, β- and γ-MnO_2_, and 2D δ-MnO_2_) owing to an octahedral [MnO_6_] spatial arrangement [47,48]. Nano-sized MnO_2_ exhibits commendable benefits, due to a larger surface-to-volume ratio and further reactive surface for electrochemical reactions. The diverse application of this substance in electrochemistry and sensor fabrication can be attributed to the simple reduction of MnO_2_ to Mn_2_O_3_ and MnO and, at the proper potential, the re-oxidation to MnO_2_ as a catalytic circle for electrochemical detection [49,50,51].

Electroanalysis and electrochemistry have recently benefited greatly from room temperature ionic liquids (RTILs), which have astonishing physicochemical properties such as an impressive thermal and chemical stability, ionic architecture, proper extraction capacity, low equilibrium vapor pressure, ion exchange activity, potent conductivity and broad electrochemical window. ILs have a better conductivity than paraffin oil, which makes them suitable for making paste electrodes in voltammetric determinations due to a reduced charge current and consequent intensification of sensitivity and a decrease in the LOD value [52,53].

Accordingly, the present study utilized these two compounds to modify the CPE surface.

The current work introduced a convenient single-phase hydrothermal protocol to fabricate MnO_2_ NR. Then, IL and MnO_2_ NR were utilized to modify the CPE surface to voltammetrically sense the SAA. An enhanced voltammetric sensitivity was found for the as-developed modified electrode toward SAA when compared with the bare CPE. The ability of the modified electrode for use in sensor applications was verified in the determination of SAA present in the real specimens, with an impressive relative standard deviation (RSD).

## 2. Materials and Methods

### 2.1. Chemicals and Equipment

An X-ray diffractometer (Panalytical X’Pert Pro, Almelo, The Netherlands) using copper/Kα radiation (λ = 1.5418 Å) captured XRD patterns. A Tensor II spectrometer (Bruker, Denkendorf, Baden-Württemberg, Germany) was utilized to record the FT-IR spectra. A scanning electron microscope (MIRA3, Tescan, Brno, Czech Republic) provided the FE-SEM images and the EDX patterns. An autolab potentiostat/galvanostat (PGSTAT-302N, Eco Chemie, Utrecht, The Netherlands) recorded all electrochemical determinations. The General Purpose Electrochemical System (GPES) as selected software monitored all testing protocols. A conventional three-electrode system was used at 25 ± 1 °C. An Ag/AgCl/KCl (3.0 M) electrode, a platinum wire and MnO_2_NR-IL/CPE were used as the reference, auxiliary and working electrodes, respectively. A pH meter (Metrohm type 713) was utilized to determine all solution pH values. All solutions were prepared freshly by deionized water (DIW, Millipore Direct-Q^®^ 8 UV water purification system, Darmstadt, Germany). Sulfanilamide, 1-butyl-3-methylimidazolium hexafluorophosphate (BMIM-PF_6_) ionic liquid, and all materials were purchased from Merck (Darmstadt, Germany) with analytical research purity. Orthophosphoric acid as well as relevant salts have been applied to achieve all phosphate buffer solutions (PBS), set in a pH range of 2.0–9.0.

### 2.2. Hydrothermal Synthesis of MnO_2_ NRs

The MnO_2_ NRs were obtained by dissolving KMnO_4_ (0.316 g) in deionized water (30 mL) while vigorously stirring, followed by the addition of 3 M HCl (1.4 mL) under vigorous stirring for another half hour. Then, the solution was placed in a 50-mL Teflon-lined autoclave at 160 °C for six hours. Next, the products were cooled down to room temperature and subsequently centrifuged and thoroughly rinsed with ethanol and deionized water to clean any impurity, followed by drying at 60 °C for 12 h.

### 2.3. Process of Electrode Fabrication

As the composition of the carbon paste is very important in electrochemical responses, we optimized all of them. To produce an MnO_2_ NR and ionic liquid-modified carbon paste electrode (MnO_2_NR-IL/CPE), firstly, ionic liquid (0.8 mL), MnO_2_ NR (0.4 g), and graphite powder (0.9 g) were blended well together in a mortar to achieve a uniformly wetted paste. Then, the paste was compressed into the bottom of a glass tube. A copper wire was embedded into the back of the mixture down in a glass tube to establish an electrical contact. Next, excess paste, if present, was expelled from the tube, and a weighing paper was used for polishing to achieve a new surface.

## 3. Results and Discussion

### 3.1. Determination of Characteristics

**Figure 1:**
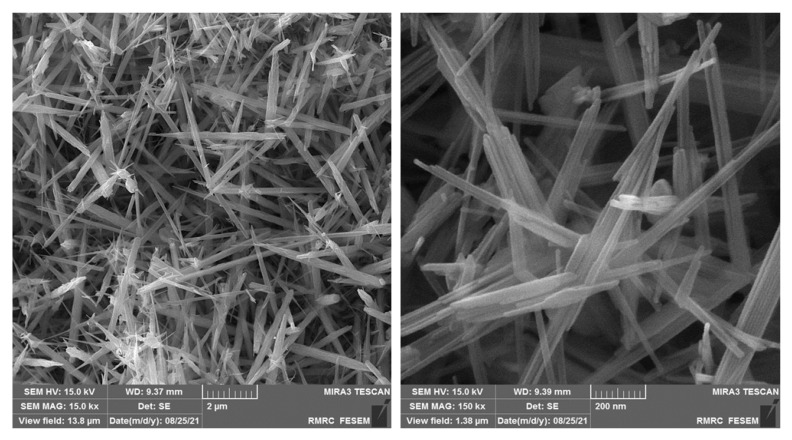
FE-SEM images captured for as-fabricated manganese dioxide nanorods, with various magnifications.

Figure 1 illustrates the FE-SEM images captured for the as-fabricated MnO_2_ NRs, and observing them confirmed rod-shaped MnO_2_ nanostructures with a thickness ranging from 15 to 25 nm and a length of about 3 μm. The MnO_2_ NRs showed an almost uniform size distribution.

**Figure 2:**
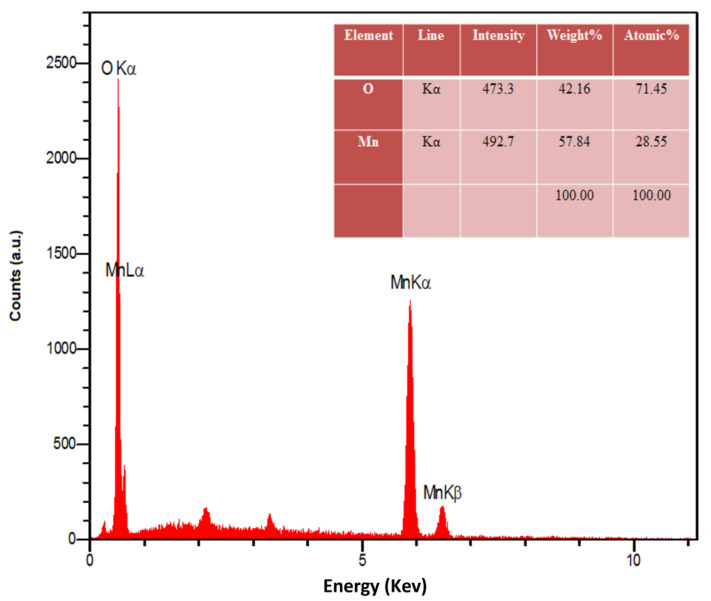
EDX spectrum captured for as-fabricated manganese dioxide nanorods.

The EDS analysis was performed to check the elemental composition of the MnO_2_ NRs. Figure 2 shows the presence of O (42.16 wt%) and Mn (57.84 wt%) in the structure of MnO_2_ NRs without any impurity.

**Figure 3:**
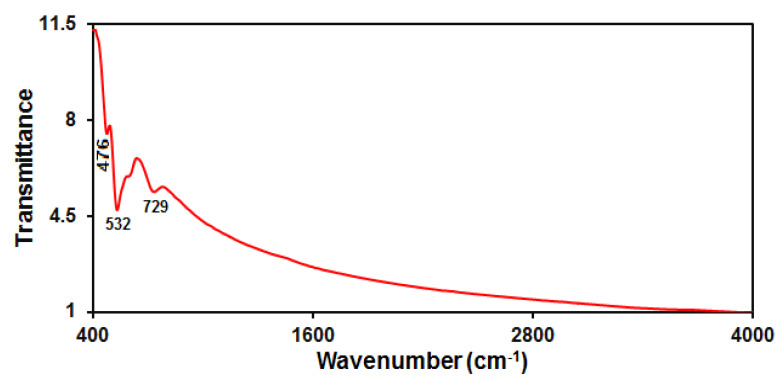
FT-IR pattern captured for as-fabricated manganese dioxide nanorods.

Figure 3 displays the FT-IR spectrum captured for as-fabricated MnO_2_ NRs, with the characteristic peaks at 476, 532 and 729 cm^−1^ corresponding to Mn-O α-phase vibration. The FT-IR spectrum of the as-prepared sample was consistent with the previous report [54].

**Figure 4:**
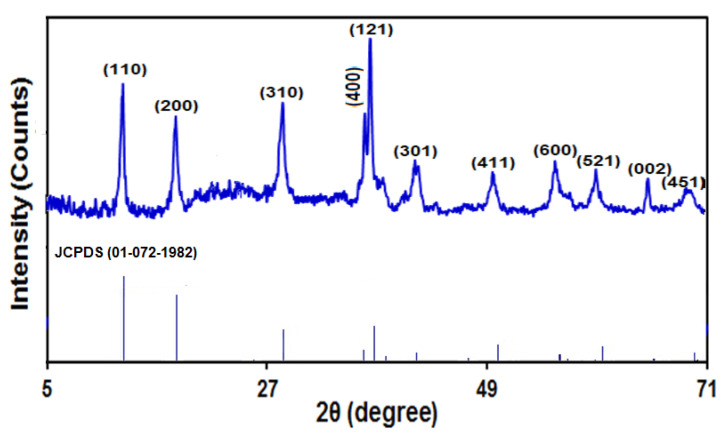
XRD spectrum captured for as-fabricated manganese dioxide nanorods.

Figure 4 portrays the XRD spectrum captured for MnO_2_ NRs, presenting a clear crystallinity at sharp intense peaks. There were peaks with corresponding planes at 12.7 (110), 18 (200), 28.7 (310), 36.5 (400), 37.6 (121), 42 (301), 49.8 (411), 56.1 (521), 60.2 (002), 65.5 (451) and 69.6°. The diffraction peaks of MnO_2_ related to a tetragonal architecture with the α phase, with ref. code 01-072-1982.

### 3.2. Electrochemical Response of SAA on the MnO_2_NR-IL/CPE Surface

**Figure 5:**
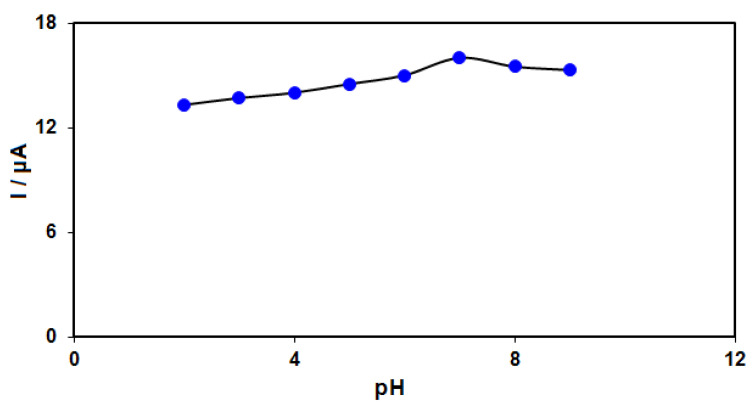
Plot of Ip vs. pH obtained from DPVs of MnO_2_NR-IL/CPE in a solution containing 50.0 μM of SAA in 0.1 PBS with different pHs (2.0, 3.0, 4.0, 5.0, 6.0, 7.0, 8.0 and 9.0).

According to the obtained findings, the SAA electro-oxidation was based on electron and proton exchange. Moreover, it was crucial to optimize the pH value in detecting the analyte. Hence, the DPV technique was applied to clarify the electrochemical response of SAA on the MnO_2_NR-IL/CPE surface in PBS (0.1 M) at various pH values between 2.0 and 9.0. Reportedly, the SAA electro-oxidation on the MnO_2_NR-IL/CPE surface was higher in neutral conditions than in alkaline or acidic medium (Figure 5). Consequently, the optimal pH was chosen as 7.0 for SAA electro-oxidation on the MnO_2_NR-IL/CPE surface.

**Figure 6:**
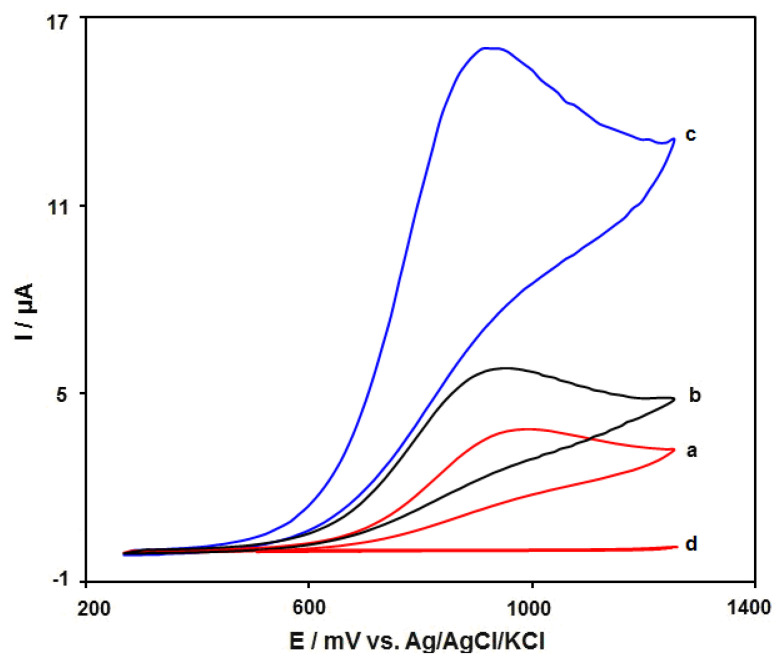
Cyclic voltammograms captured for (**a**) bare CPE, (**b**) MnO_2_NR/CPE and (**c**) MnO_2_NR-IL/CPE in PBS (0.1 M) at the optimized pH value of 7.0 under exposure to 50.0 μM of SAA at a scan rate of 50 mV/s. Additionally, (**d**) as (**c**) in the absence of SAA.

The electrochemical performance of MnO_2_NR-IL/CPE in comparison with other modified electrodes was studied by the cyclic voltammetry (CV) technique under exposure to 50.0 μM of SAA at 50 mV/s in PBS (0.1 M). The CVs of all as-fabricated electrodes in this study can be observed in Figure 6. The MnO_2_NR-IL/CPE voltammetric behavior (Figure 6c) exhibited the relatively strongest oxidation peak at 925 mV, with the oxidation peak current at 16.0 μA, sequentially. The MnO_2_NR/CPE voltammetric behavior (Figure 6b) exhibited the relatively strongest oxidation peak at 960 mV, with the oxidation peak current at 9.3 μA, sequentially, and the bare CPE voltammetric behavior (Figure 6a) exhibited a relatively weak oxidation peak with less intensity at 1000 mV, with the oxidation peak current at 3.8 μA, sequentially. Consequently, the MnO_2_NR-IL/CPE has an obviously better electrocatalytic behavior towards the SAA with a relatively strong current response. Additionally, bare in PBS without SAA did not show any peak.

### 3.3. Effect of Scan Rate

**Figure 7:**
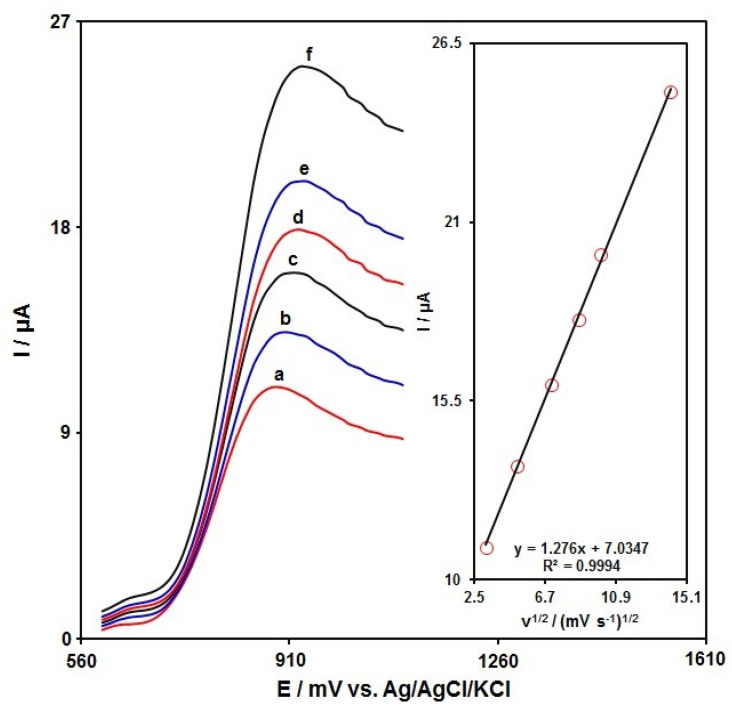
LSV captured for MnO_2_NR-IL/CPE in PBS (0.1 M) at the optimized pH value of 7.0 under exposure to 5.0 μM of SAA at various scan rates; the curves nos. a to f correspond to 10, 25, 50, 75, 100 and 200 mV/s, sequentially; Inset: changes in anodic peak currents against v^1/2^.

The scan rate performance on the SAA oxidation peak current was investigated to analyze electrode processes. The linear sweep voltammograms (LSVs) were recorded for 50.0 μM of SAA in PBS (0.1 M) at various scan rates, and the results indicated that anodic peak currents (Ipa) were linearly related to the scan rate square root (Figure 7). Accordingly, the diffusion-controlled process was seen for the SAA oxidation on the MnO_2_NR-IL/CPE.

**Figure 8:**
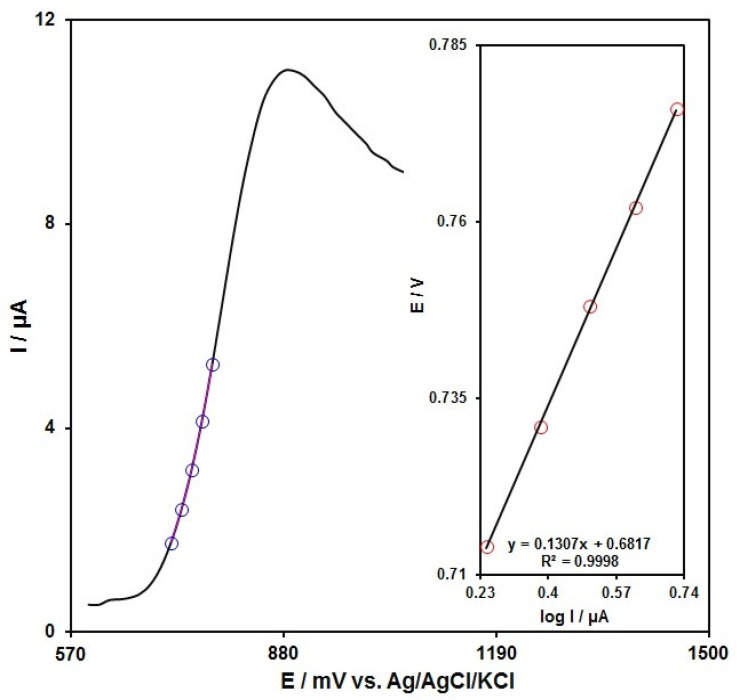
LSV captured for MnO_2_NR-IL/CPE in PBS (0.1 M) at the optimized pH value of 7.0 under exposure to 50.0 μM of SAA at 10 mV/s; Points: information applied in the Tafel plot; Inset: LSV-derived Tafel plot.

Tafel graph was plotted to achieve data on the rate-determining step, as obtained from points of the Tafel area in linear sweep voltammetry. Figure 8 illustrates the Tafel plot for 50.0 μM SAA oxidation in PBS (0.1 M) on the MnO_2_NR-IL/CPE surface.

In this condition, the transfer coefficient (α) can be estimated from the slope of the Tafel plot:

η = b log i + constant

where b = 2.3RT/(1 – α)nF.

An average slope of 0.1307 V is obtained, indicating a one-electron transfer for a rate-limiting step assuming a transfer coefficient of α = 0.55.

### 3.4. Chronoamperometric Determinations

**Figure 9:**
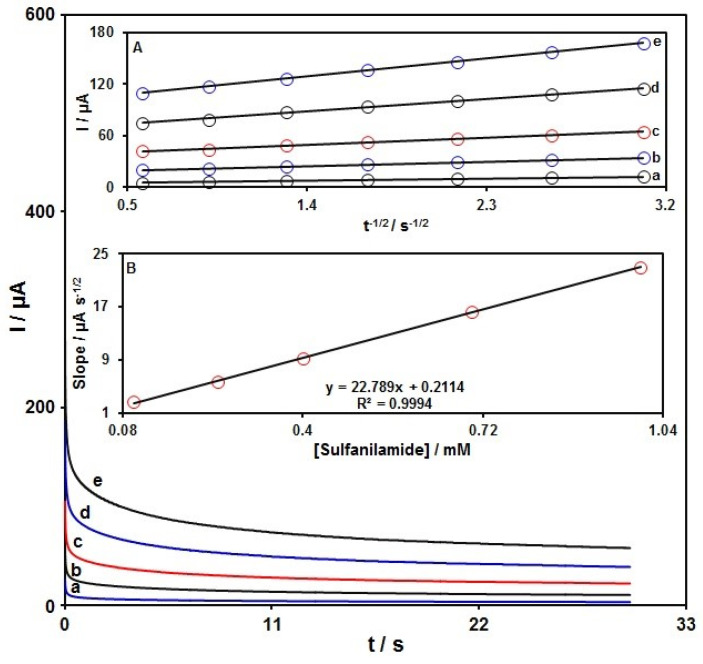
Chronoamperograms for MnO_2_NR-IL/CPE in PBS (0.1 M) at the optimized pH 7.0 for different SAA contents; the curves nos. a to e correspond to 0.1, 0.25, 0.4, 0.7 and 1.0 mM of SAA; Insets: (**A**) Plots of I against t^−1/2^ from chronoamperograms a to e. (**B**) Plot of straight lines slope against SAA content.

Figure 9 shows the chronoamperometric analysis to calculate the diffusion coefficient of SAA. The plots of I against t^−1/2^ have been utilized in relation to the optimal fits for different SAA contents in PBS (0.1 M), as seen in Figure 9A. The chronoamperometric measurements were carried out for various SAA contents on MnO_2_NR-IL/CPE at the working electrode potential of 975 mV. The slopes of the obtained straight lines versus SAA contents were drawn by the Cottrell equation in order to obtain the below equation:

I = nFAD^1/2^ C_b_π^−1/2^t^−1/2^

with D (cm^2^/s) being the diffusion coefficient, n the number of electrons for oxidation of SAA (n = 1) and C_b_ (mol/cm^3^) the bulk content. There was a linearity for the I against t^−1/2^ plot under diffusion control over various SAA contents. The slopes could be used to compute the mean D value for SAA (see Figure 9B), which was 2.2 × 10^−5^ cm^2^/s.

### 3.5. Detection Limit and Standard Plot

**Figure 10:**
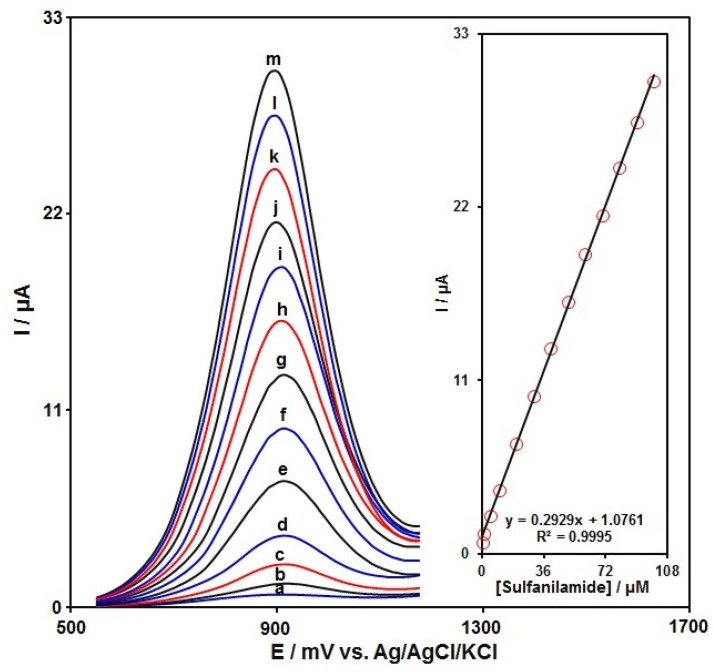
DPVs captured for MnO_2_NR-IL/CPE in PBS (0.1 M) at the optimized pH of 7.0 under exposure to various SAA contents; nos. a to m correspond to 0.07, 0.7, 5.0, 10.0, 20.0, 30.0, 40.0, 50.0, 60.0, 70.0, 80.0, 90.0 and 100.0 μM of SAA; Inset: plot of peak current as a function of various SAA contents (0.07–100.0 μM).

The SAA content was measured by the DPV technique. The DPVs captured for MnO_2_NR-IL/CPE at various SAA contents in PBS (0.1 M) are shown in Figure 10. There was a stepwise enhancement in the SAA oxidation current by gradually increasing the SAA contents, which is to say the applicability of MnO_2_NR-IL/CPE for electrochemically sensing the SAA. Figure 10 (inset) represents the alterations in the oxidation signal for MnO_2_NR-IL/CPE as a function of various SAA contents (0.07–100.0 μM), showing a low detection limit of 0.01 μM. Moreover, Table 1 shows that MnO_2_NR-IL/CPE can be successfully used for the determination of SAA, compared with other sensors.

### 3.6. Interference Studies

Interference studies were investigated to know how the results for the SAA analysis were affected by the presence of various compounds. According to the used definition, the tolerance limit was defined as the ratio of the concentration of the interfering compounds to the SAA, which led to a relative error of less than ±5.0%. The possible interference was investigated by the addition of various compounds such as Mg^2+^, Na^+^, K^+^, Ca^2+^, Cl^−^, ascorbic acid, uric acid, glucose, sucrose, L-cystine, and dopamine to PBS (pH 7.0) in the presence of 50.0 μM SAA. It was found that the addition of these interfering species had no remarkable effect on the DPV signal of SAA. These results indicate that the modified electrode has a good selectivity for SAA determination.

### 3.7. Real Sample Analysis

The ability of MnO_2_NR-IL/CPE to be used for sensor applications in the detection of SAA was determined in real specimens of urine and water according to the standard addition method, as seen in Table 2. The recorded recovery rates ranged from 97.0% to 104.2%, thereby highlighting the appreciable applicability of MnO_2_NR-IL/CPE in sensing the SAA in real specimens.

## 4. Conclusions

A facile hydrothermal method was applied to prepare uniform and pure manganese dioxide NRs. Then, ionic liquid and MnO_2_ NRs were utilized to modify a carbon paste electrode surface (MnO_2_NR-IL/CPE) to voltammetrically sense the SAA. An enhanced electrochemical performance was found for the as-developed modified electrode toward SAA oxidation when compared with a bare electrode, with a lengthy linear range (0.07–100.0 μM) and a narrow limit of detection (0.01 μM). The as-fabricated sensor possessed an exceptional electrocatalytic behavior, excellent sensitivity and low limit of detection. Additionally, the MnO_2_NR-IL/CPE showed a good selectivity for the detection of SAA in the presence of various compounds. The ability of the modified electrode to be used for sensor applications was verified by the determination of SAA present in actual human urine and serum specimens, with impressive recovery outcomes.

## Figures and Tables

**Table 1:** Comparison of the linear range and detection limit obtained for MnO_2_NR-IL/CPE with the determination of SAA with other sensors.

Electrochemical Sensor	Method	Linear Dynamic Range	Limit of Detection	Ref.
Au nanoparticle-functionalized graphene/glassy carbon electrode	DPV	0.1–1000 μM	0.011 μM	[55]
Nanodiamond/glassy carbon electrode	Square wave voltammetry	1.2–581.4 μM	0.94 μM	[56]
Carboxylmultiwalled carbon nanotubes/glassy carbon electrode	Cyclic voltammetrty	1–100 μM	0.5 μM	[57]
Fe_3_O_4_/functionalized Graphene/glassy carbon electrode	Amperometry	0.5–110 μM	0.05 μM	[58]
Pyrrole/molecularly imprinted polymer pencil graphite electrode	DPV	0.05–1.1 and 1.1–48 μM	0.02 μM	[59]
MnO_2_NR-IL/CPE	DPV	0.07–100.0 μM	0.01 μM	This Work

**Table 2:** Confirmed applicability of MnO_2_NR-IL/CPE in sensing the SAA in real specimens (n = 5); all concentrations are in μM.

Sample	Spiked	Found	Recovery (%)	R.S.D. (%)
Human urine	0	-	-	-
	4.5	4.6	102.2	3.3
	6.5	6.3	97.0	2.8
	8.5	8.4	98.8	1.8
	10.5	10.6	100.9	2.3
	0	-	-	-
Blood serum	5.0	4.9	98.0	2.2
	7.0	7.3	104.3	3.5
	9.0	8.9	98.9	1.9
	11.0	11.2	101.8	2.7

## Data Availability

The data presented in this study are available on request from the corresponding authors.
